# Coinfections With Respiratory Syncytial Virus in a Cohort of Adults With High‐Risk Conditions, 2015–2020: A Retrospective Observational Study

**DOI:** 10.1002/hsr2.73418

**Published:** 2026-11-03

**Authors:** Oluwakemi D. Alonge, Maria E. Sundaram, Huong Q. Nguyen, Jennifer P. King, Elisha Stefanski, Andrew G. Allmon, Jean‐Yves Pirçon, Pouya Saeedi

**Affiliations:** ^1^ Center for Clinical Epidemiology and Population Health, Marshfield Clinic Research Institute Marshfield Wisconsin USA; ^2^ Integrated Research and Development Laboratory, Marshfield Clinic Research Institute Marshfield Wisconsin USA; ^3^ GSK Durham North Carolina USA; ^4^ GSK Wavre Belgium

**Keywords:** adults, coinfections, high‐risk conditions, respiratory syncytial virus

## Abstract

Coinfections may influence the clinical outcome of respiratory illness. Data are limited regarding RSV coinfections in adults with high‐risk conditions. Residual respiratory specimens from community‐dwelling adults (≥ 18 years) with ≥ 1 high‐risk condition were retested using a multiplex panel for multiple viral pathogens. We compared demographic and clinical characteristics between those with RSV coinfection versus RSV single infection. RSV coinfections comprised 5.9% of all RSV infections among adults with ≥ 1 high‐risk condition. No statistically significant differences were observed between those with RSV coinfections and single infections according to presence of specific high‐risk conditions or clinical disease presentation.

## 1 Introduction

Coinfections with respiratory syncytial virus (RSV) and other respiratory pathogens may affect symptoms and disease severity [1, 2]. RSV coinfections in children are associated with increased disease severity, including pneumonia, severe bronchiolitis, and hospitalization [3]. However, there are limited data on the significance of RSV coinfections in adults with high‐risk conditions (“high‐risk adults”), who may be at increased risk for severe infection. We identified RSV coinfections among high‐risk adults during five winter seasons, and compared demographic and clinical characteristics between those with RSV coinfections versus single infections.

## 2 Methods

This retrospective cohort study used existing data and archived respiratory specimens from community‐dwelling adult participants who were originally enrolled in test‐negative studies of influenza vaccine effectiveness (VE) during 2015–2020 winter seasons. The details of these VE studies have been described in detail elsewhere [4] and are summarized briefly below.

### 2.1 Influenza Vaccine Effectiveness Study

Original VE studies enrolled Marshfield Clinic Health System (MCHS) patients in Marshfield, WI, concordant with an outpatient clinic encounter for an acute respiratory illness (ARI), during winter seasons (January 2016–April 2016, January 2017–April 2017, December 2017–April 2018, January 2019–April 2019, December 2019–March 2020). Eligible participants had illness duration of ≤ 7 days at the time of enrollment. Individuals with enrollment in the past 14 days or receipt of an antiviral drug before sample collection were ineligible. Participants provided respiratory samples (nasal and throat) for influenza testing. Residual samples were subsequently archived.

### 2.2 Current Study

The current analysis included VE study participants aged ≥ 18 years with ≥ 1 high‐risk condition. Archived respiratory samples were retested on a multiplex panel (GenMark ePlex Respiratory Pathogen Panel) [5] for adenovirus (AdV), seasonal coronaviruses (CoV; types 229E, HKU1, NL63, OC43), human metapneumovirus (hMPV), rhinovirus/enterovirus (RV), influenza virus (IFV; A/H1, A/H1pdm09, H3, B), parainfluenza virus (PIV; types 1–4), and RSV (types A, B). RSV coinfection was defined as detection of ≥ 2 viral pathogens, one of which was RSV A or B and the other non‐RSV. Infection with RSV A and B simultaneously, without the presence of a non‐RSV pathogen, was not considered a coinfection. High‐risk conditions were defined using International Classification of Diseases (ICD) codes documented in the electronic health record (EHR) (Appendix A). Conditions were grouped into cardiac disorders, chronic respiratory diseases, chronic liver disease, chronic kidney disease, diabetes, and immunocompromised status. Charlson Comorbidity Index (CCI) score at time of VE study enrollment, and hospitalization within 30 days of enrollment visit, were also obtained from the EHR.

### 2.3 Ethics Statement

The MCHS institutional review board reviewed and approved this study with a waiver of informed consent. Patients provided consent before participating in the original Flu VE study.

## 3 Statistical Analysis

The prevalence of RSV coinfection was calculated as the proportion of enrollments with RSV A or B and ≥ 1 non‐RSV pathogen(s), among total RSV‐positive enrollments. Prevalence of RSV single infection was calculated as the proportion of enrollments with RSV A or B only, among total RSV‐positive enrollments. Differences in demographic and clinical characteristics between RSV coinfections versus single infections were assessed using two‐tailed chi‐square, Fisher's exact, or Wilcoxon rank‐sum tests, as appropriate. Absolute differences in proportions and Wald 95% confidence intervals (CIs) between groups were calculated; *p* < 0.05 were considered statistically significant. Analyses were performed using Stata v19 (College Station, TX) and SAS v9.4 (Cary, NC). Multivariable analyses were not conducted due to sparse analytic strata. The MCHS IRB reviewed and approved this study with a waiver of informed consent.

## 4 Results

Of 3601 samples retested from eligible enrollments of high‐risk adults, RSV was detected in 303 (8.4%). Among RSV‐positive samples, 39.6% were from adults aged ≥ 65 years, 65.7% from females, and 69.6% from those who received influenza vaccine in the past year (Table 1).

**Table 1:** Demographic and clinical characteristics among adults with high‐risk conditions who had RSV single infections and RSV coinfections from 2015 to 2020 respiratory virus winter seasons.

	Total respiratory samples tested from adults with high‐risk conditions^a^		RSV single infection	RSV coinfection	*P*‐value for comparison between RSV coinfection and single infection	Absolute difference in proportions: (RSV coinfection/total RSV) – (RSV single infection/total RSV)
		Total RSV‐positive^b^				
		*N* (%)	*N* (%)	*N* (%)		% (95% CI)
Overall: *N*	3601	303	285 (94.1)	18 (5.9)		88.1 (84.4–91.9)
Age group						
18–49 years	1457	90 (29.7)	81 (28.4)	9 (50.0)	0.06	21.6 (−2.1 to 45.3)
50–59 years	697	58 (19.1)	53 (18.6)	5 (27.8)	0.35	9.1 (−12 to 30.4)
60–64 years	367	35 (11.6)	34 (11.9)	1 (5.6)	0.71	6.4 (−4.9 to 17.6)
≥ 65 years	1080	120 (39.6)	117 (41.1)	3 (16.7)	0.05	24.3 (6.3 to 42.5)
Sex						
Male	1243	104 (34.3)	95 (33.3)	9 (50.0)	0.19	16.7 (−7.1 to 40.4)
Female	2358	199 (65.7)	190 (66.7)	9 (50.0)	0.19	16.7 (−7.1 to 40.4)
Vaccination status						
Received influenza vaccine in the past year	2203	211 (69.6)	201 (70.5)	10 (55.6)	0.19	14.9 (−8.6 to 38.5)
Received pertussis vaccine ever	3427	288 (95.0)	271 (95.1)	17 (94.4)	0.90	0.6 (−10.2 to 11.5)
Received pneumococcal vaccine ever	2135	206 (67.9)	196 (68.8)	10 (55.6)	0.29	13.2 (−10.4 to 36.8)
Illness signs and symptoms^c^						
Fever/feverishness	2303	168 (55.4)	157 (55.1)	11 (61.1)	0.62	6.0 (−17.2 to 29.3)
Fatigue/feeling run down	3365	283 (93.4)	266 (93.3)	17 (94.4)	0.85	1.1 (−9.9 to 12.1)
Nasal congestion	2962	278 (91.7)	262 (91.9)	16 (88.9)	0.65	3.0 (−11.8 to 17.9)
Wheezing	2207	227 (74.9)	214 (75.1)	13 (81.3)	0.79	6.2 (−13.6 to 25.9)
Shortness of breath/trouble breathing	2392	222 (73.3)	212 (74.4)	10 (55.6)	0.09	18.8 (−4.7 to 42.3)
Sore throat	2418	195 (64.4)	182 (63.9)	13 (72.2)	0.47	8.4 (−13.1 to 29.8)
Muscle pain/myalgia^c^	580	42 (13.9)	38 (13.3)	4 (22.2)	0.29	8.9 (−10.7 to 28.5)
Headache^c^	634	48 (15.8)	44 (15.4)	4 (22.2)	0.50	6.8 (−12.9 to 26.4)
Vomiting^c^	301	27 (8.9)	24 (8.4)	3 (16.7)	0.21	8.3 (−9.3 to 25.8)
Hospitalization in the 30 days after study enrollment^d^	96	8 (2.6)	8 (2.6)	0	—	—
30‐day post‐infection asthma exacerbation^e^	140	12 (3.9)	10 (3.5)	2 (11.1)	0.15	7.6 (−7.1 to 22.3)
30‐day post‐infection COPD exacerbation^e^	142	20 (6.6)	19 (6.7)	1 (5.6)	*p* > 0.99	1.1 (−9.9 to 12.1)
30‐day post‐infection pneumonia^f^	156	14 (4.6)	13 (4.6)	1 (5.6)	0.58	1.0 (−9.9 to 11.9)
High‐risk conditions^g^						
Any cardiac disorder^h^	2470	211 (69.6)	200 (70.2)	11 (61.1)	0.43	9.1 (−14.1 to 32.2)
Any chronic respiratory disease^i^	1568	140 (46.2)	132 (46.3)	8 (44.4)	*p* > 0.99	1.9 (−21.8 to 25.6)
Chronic liver disease	494	49 (16.2)	46 (16.1)	3 (16.6)	*p* > 0.99	0.5 (−17.2 to 18.3)
Chronic kidney disease	1109	104 (34.3)	98 (34.4)	6 (33.3)	*p* > 0.99	1.1 (−21.4 to 23.5)
Diabetes	908	87 (28.7)	82 (28.8)	5 (27.8)	*p* > 0.99	1.0 (−20.4 to 22.3)
Immunocompromised^j^	226	27 (8.9)	25 (8.8)	2 (11.1)	0.67	2.3 (−12.6 to 17.2)

*Note:* 95% confidence intervals were calculated for the absolute difference in demographic and clinical characteristics between those with RSV single infection versus RSV coinfection. Difference is statistically significant when the confidence interval does not include 0.

^a^ It is possible for individuals to have been enrolled more than once in a given respiratory virus season, provided illnesses were at least 14 days apart. However, there were no individuals with more than one study‐identified RSV‐positive ARI in the same respiratory virus season.

^b^ RSV coinfection was identified in 5.9% of RSV‐positive adults and RSV single infection in 94.1%.

^c^ Participants were asked about these signs and symptoms at enrollment during each season of the original test‐negative design study. Information regarding muscle pain/myalgia, headache, and vomiting was only collected during the 2019–20 winter respiratory virus season. Percentages for muscle pain/myalgia, headache, and vomiting are calculated using denominators restricted to the 2019–20 respiratory virus season, where 67 RSV‐positive cases were observed (6 RSV coinfections and 61 RSV single infections).

^d^ We originally identified 10 hospitalizations among study participants with RSV single infection. However, upon further manual chart review, we identified 2 hospitalizations that were ruled out.

^e^ An exacerbation was defined as either an ICD‐10 code indicating exacerbation, or as a “hospitalization, urgent care, or emergency department visit within 30 days following a medically attended acute respiratory illness (MAARI), where the diagnosis at admission/intake or discharge refers to the underlying condition”. In these instances, visits to a primary care clinic would not be included. We chose to exclude these visits from the analysis instead of categorizing them as ‘non‐exacerbation’ or ‘exacerbation’.

^f^ Defined as pneumonia onset in the 30 days after symptom onset if they have at least one ICD code relating to pneumonia following symptom onset preceding a specific MAARI visit, plus a code indicating the presence of a chest X‐ray.

^g^ Cardiac disorders, chronic respiratory disease, chronic liver disease, chronic kidney disease, diabetes, and ‘immunocompromised’ definitions are not mutually exclusive. For example, it is possible for an individual to have both a cardiac disorder and an immunocompromising condition.

^h^ Cardiac disorders include arrhythmias, heart failure, and coronary artery disease.

^i^ Chronic respiratory diseases include COPD, asthma, and cystic fibrosis.

^j^ Immunocompromising conditions include individuals with transplants (not restricted to solid organ transplant), malignancies, immunosuppressive therapy, and/or other immunodeficiencies.

Among RSV‐positive samples, 18 (5.9%) were coinfections and 285 (94.1%) were single infections. Prevalence of RSV coinfections varied by age, and occurred with RV (*n* = 8; 44.4%); IFV (*n* = 6; 33.3%); CoV (*n* = 5; 27.8%); and AdV (*n* = 1; 5.6%) (Figure 1). Demographic characteristics and high‐risk conditions among those with RSV single versus coinfections are shown in Table 1.

**Figure 1:**
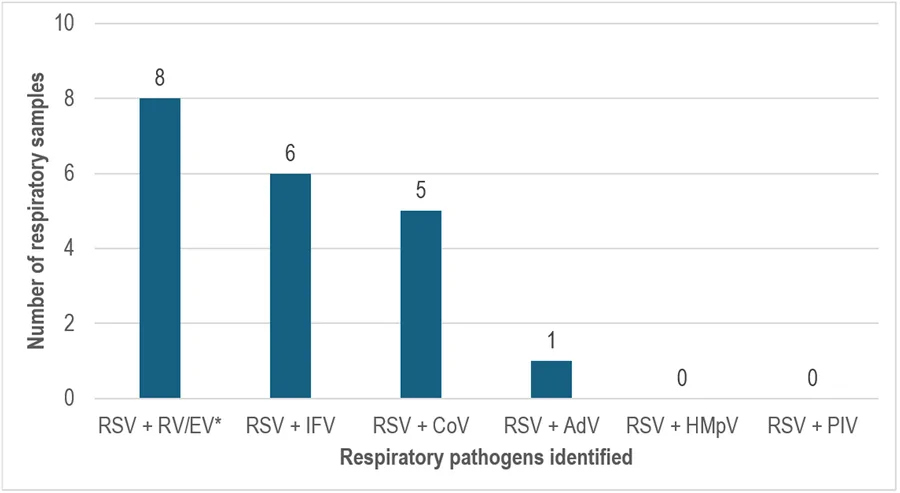
Distribution of RSV viral‐viral coinfections by number of respiratory samples tested. *Two respiratory samples were positive for RSV + HRV/HEV + IFV. AdV, adenovirus; CoV, coronaviruses (seasonal); EV, enterovirus; HMpV, human metapneumovirus; IFV, influenza virus; PIV, parainfluenza virus; RSV, respiratory syncytial virus. RV, rhinovirus

In the 30 days after enrollment, there were 0 hospitalizations among RSV coinfections and 8 hospitalizations (2.6%) among RSV single infections. During this period, RSV‐coinfected individuals experienced 2 (11.1%), 1 (5.6%) and 1 (5.6%) cases of asthma exacerbation, chronic obstructive pulmonary disease (COPD) exacerbation, and pneumonia, respectively. Among RSV single infections, there were 10 (3.5%), 19 (6.7%) and 13 (4.6%) cases with asthma exacerbation, COPD exacerbation, and pneumonia, respectively. There were no statistically significant differences between those with RSV coinfections versus single infections by age group, sex, vaccination status, presence of upper or lower respiratory symptoms, or high‐risk conditions.

## 5 Discussion

In this retrospective cohort study of high‐risk adults, RSV coinfections comprised 5.9% of all RSV outpatient ARI. The relatively low proportion of RSV coinfections observed among high‐risk adults in our study is consistent with previous studies of hospitalized children and adults, where respiratory viral coinfections (including coinfections with RSV) varied from 5.0% to 14.4%, and were inversely associated with age [6, 7]. RSV coinfection was most common with RV and IFV in our study, consistent with other studies that have reported higher proportions of RSV/RV in adult coinfections [8, 9].

Existing literature has reported a higher proportion and incidence of RSV infections versus no infection in adults with cardiac disorders compared to other comorbidities, as well as increased morbidity and mortality due to RSV infections in general among adults with underlying COPD or congestive heart failure (CHF) compared to adults without comorbidities [10, 11, 12]. We identified no statistically significant differences between RSV coinfections and RSV single infections according to high‐risk conditions, potentially as a result of limited statistical power.

Our study had several limitations. This analysis had a relatively small sample size and low number of RSV coinfections detected, limiting our ability to detect differences in coinfection and single‐infection groups. Additionally, we were limited in our ability to detect coinfections by the pathogens present in the multiplex panel, though the panel included common viral respiratory pathogens. Our study period was before the COVID‐19 pandemic, and does not include coinfections with RSV and SARS‐CoV‐2. Furthermore, our analysis is limited to ARI identified in the outpatient setting. As such, it necessarily excludes high‐risk patients who sought care only in the hospital or emergency department. This limits generalizability of our findings. Although our identification of high‐risk conditions is in keeping with other studies on RSV in adults, there is likely to be heterogeneity in severity of comorbid conditions within high‐risk groups. Many epidemiological assessments of RSV in high‐risk adults have focused on hospitalized cohorts and report clinical outcomes of severe illness, such as hospitalization, ICU admission, and death [11, 13]. Finally, our study population was predominantly non‐Hispanic White, which may limit the generalizability of study findings.

In a multiseason study of high‐risk adults with ARI, we found that RSV coinfections are relatively uncommon in the outpatient setting, and do not differ significantly from RSV single infection by high‐risk conditions or disease presentation. There is a need for ongoing research to assess the burden of RSV coinfections in adults with high‐risk conditions who are most likely to benefit from RSV vaccination.

## Author Contributions

**Oluwakemi D. Alonge:** conceptualization, writing – original draft, methodology, investigation, visualization, writing – review and editing, formal analysis, data curation, validation. **Maria E. Sundaram:** conceptualization, funding acquisition, methodology, writing – review and editing, data curation, supervision, formal analysis, investigation. **Huong Q. Nguyen:** writing – review and editing, conceptualization, methodology. **Jennifer P. King:** writing – review and editing, conceptualization, methodology. **Elisha Stefanski:** validation, writing – review and editing. **Andrew G. Allmon:** writing – review and editing. **Jean‐Yves Pirçon:** conceptualization, methodology, writing – review and editing. **Pouya Saeedi:** conceptualization, methodology, writing – review and editing.

## Conflicts of Interest

O.D.A., M.E.S., H.Q.N., J.P.K. and E.S. are employed by Marshfield Clinic Research Institute, which received funding from GSK to support the independent scientific work reported in this publication. O.D.A., H.Q.N. and J.P.K. receive research support from CSL Seqirus and ModernaTX. A.G.A., J.‐Y.P. and P.S. are employees of GSK; A.G.A., J.‐Y.P. and P.S. hold financial equities in GSK.

## Transparency Statement

Ms. Alonge, as the lead author, affirms that this manuscript is an honest, accurate, and transparent account of the study being reported; that no important aspects of the study have been omitted; and that any discrepancies from the study as planned have not occurred.

## Data Availability

The data that support the findings of this study are available on request from the corresponding author. The data are not publicly available due to privacy or ethical restrictions.

## Appendix A

See Table A1.

**Table A1:** ICD‐10 codes used to identify an individual as having a high‐risk condition.

Condition^a^	ICD‐10 code	Time frame^b^	ICD‐9 codes (if needed)
**Chronic respiratory diseases**			
Aspergillosis	B44*	Past 5 years	117.3
Cystic fibrosis	E84*	Ever	277.01, 277.02, 277.03
Simple and mucopurulent chronic bronchitis	J41 (J41.0, J41.1, J41.8)	Ever	491.0, 491.1, 491.8
Unspecified chronic bronchitis	J42*	Ever	491.9
Emphysema	J43*	Ever	492.0, 492.8
MacLeod syndrome	J43.0	Ever	492.8
Panlobular emphysema	J43.1	Ever	492.8
Centrilobular emphysema	J43.2	Ever	492.8
Other emphysema	J43.8	Ever	492.8
Emphysema, unspecified	J43.9	Ever	492.0, 492.8
Other chronic obstructive pulmonary disease	J44 (J44.0, J44.1, J44.9)	Ever	491.22, 493.21, 491.21, 493.22, 491.20, 493.20, 496
Asthma	J45*	Past 5 years	493.00–493.99
Bronchiectasis	J47*	Ever	494.0, 494.1
Coalworker's pneumoconiosis	J60*	Ever	500
Pneumoconiosis due to asbestos and other mineral fibers	J61*	Ever	501
Pneumoconiosis due to dust containing silica	J62*	Ever	502
Aluminosis (of lung), bauxite fibrosis (of lung), berylliosis, graphite fibrosis (of lung), siderosis, and stannosis	J63.0–63.5	Ever	503
Farmer's lung, bagassosis, bird fancier's lung, suberosis, maltworker's lung, mushroom‐worker's lung, maple‐bark‐stripper's lung, air conditioner and humidifier lung, and hypersensitivity pneumonitis due to other organic dusts	J67.0–67.8	Ever	495.0, 495.1, 495.2, 495.3, 495.4, 495.5, 495.6, 495.7, 495.8
Chronic respiratory conditions due to chemicals, gases, fumes and vapors	J68.4	Ever	506.4
Acute respiratory distress syndrome	J80*	Past 5 years	518.82
Pulmonary eosinophilia, not elsewhere classified	J82*	Ever	518.3
Other interstitial pulmonary diseases	J84*	Ever	515, 516.0, 516.1, 516.30, 516.31, 516.32, 516.33, 516.34, 516.37, 516.8, 516.9
Chronic respiratory failure	J96.1*	Ever	518.83
Interstitial emphysema	J98.2*	Ever	518.1
Respiratory disorders in diseases classified elsewhere	J99*	Ever	517.8
**Chronic liver disease**			
Chronic viral hepatitis	B18*	Ever	070.32, 070.33, 070.54, 070.59
Glycogen storage disease	E74.0*	Ever	271.0
Disorders of copper metabolism	E83.0*	Ever	275.1
Disorders of iron metabolism	E83.1*	Ever	275.01, 275.02, 275.03, 275.09
Alcoholic liver disease (alcoholic fatty liver, alcoholic hepatitis, alcoholic fibrosis and sclerosis of liver, alcoholic cirrhosis of liver)	K70.0–K70.3	Ever	571.0, 571.1, 571.2
Alcoholic hepatic failure	K70.4*, K70.40, K70.41	Ever	571.3
Alcoholic liver disease, unspecified	K70.9	Ever	571.3
Toxic liver disease	K71 (K71.0, K71.1, K71.10, K71.11, K71.2, K71.3, K71.4, K71.5, K71.50, K71.51, K71.6, K71.7, K71.8, K71.9)	Ever	573.3
Hepatic failure, not elsewhere classified	K72 (K72.0, K72.00, K72.01, K72.10, K72.11, K72.9, K72.90, K72.91)	Ever	570, 572.2, 572.8
Chronic hepatitis, not elsewhere classified	K73*	Ever	571.40, 571.41, 571.49
Fibrosis and cirrhosis of liver	K74.0*– K74.6*	Ever	571.5, 571.6, 571.9
Nonspecific reactive hepatitis	K75.2	Ever	573.3
Granulomatous hepatitis, not elsewhere classified	K75.3	Ever	573.3
Auto‐immune hepatitis	K75.4	Ever	571.42
Other specified inflammatory liver diseases	K75.8*	Ever	571.8, 573.3
Fatty (change of) liver, not elsewhere classified	K76.0	Ever	571.8
Hepatorenal syndrome	K76.7	Ever	572.4
**Cardiac disorders**			
Rheumatic heart failure	I09.81	Ever	398.91
Hypertensive heart disease with heart failure	I11.0	Ever	402.01, 402.11, 402.91
Angina pectoris	I20*	Between 5 years before and 6 months before index date^a^	413.0, 413.1, 413.9
Acute myocardial infarction	I21*	Between 5 years before and 6 months before index date^a^	410*
Subsequent ST elevation (STEMI) and non‐ST elevation (NSTEMI) myocardial infarction	I22*	Between 5 years before and 6 months before index date^a^	410*
Certain current complications following ST elevation (STEMI) and non‐ST elevation (NSTEMI) myocardial infarction (within the 28 day period)	I23*	Past 5 years	429.79, 429.71, 429.5, 429.6
Hypertensive heart and chronic kidney disease	I13*	Ever	404.00, 404.01, 404.02, 404.03, 404.10, 404.11, 404.12, 404.13, 404.90, 404.91, 404.92, 404.93
Other acute ischemic heart diseases	I24*	Between 5 years before and 6 months before index date^a^	411.81, 411.0, 411.89
Chronic ischemic heart disease	I25*	Ever	414.01, 414.02, 414.03, 414.04, 414.05, 414.06, 414.07, 414.10, 414.11, 414.12, 414.19, 414.2, 414.3, 414.4, 414.8, 414.9, 429.2, 412
Other pulmonary heart diseases	I27*	Ever	416.0, 416.1, 416.2, 416.8, 416.9
Cardiomyopathy	I42*	Ever	425.0, 425.11, 425.18, 425.3, 425.4, 425.5, 425.9
Atrioventricular and left bundle‐branch block	I44 (I44.0, I44.1, I44.2, I44.3, I44.30, I44.39, I44.4, I44.5, I44.6, I44.60, I44.69, I44.7)	Ever	426.11, 426.12, 426.13, 426.0, 426.10, 426.50, 426.2, 426.3
Other conduction disorders	I45*	Ever	426.4, 426.50, 426.51, 426.52, 426.53, 426.54, 426.6, 426.7, 426.81, 426.82, 426.89, 426.9
Atrial fibrillation and flutter	I48*	Ever	427.31, 427.32
Other cardiac arrhythmias	I49.1, I49.2, I49.3, I49.4, (I49.4, I49.40, I49.49), I49.5, I49.8, I49.9	Ever	427.41, 427.42, 427.61, 427.0, 427.69, 427.60, 427.69, 427.81, 427.89, 427.9
Encounter for adjustment and management of implanted device	Z45.0, Z45.01, Z45.010, Z45.018, Z45.02, Z45.09	Past 1 year	V53.31, V53.32, V53.39
Heart failure	I50*	Ever	428.0, 428.1, 428.20, 428.21, 428.22, 428.23, 428.30, 428.31, 428.32, 428.33, 428.40, 428.41, 428.42, 428.43, 428.9
Presence of cardiac and vascular implants and grafts; other postprocedural states	Z95.0, Z95.1, Z95.5, Z95.810, Z98.61	Ever	V45.01, V45.81, V45.82, V45.02
Abnormalities of heart beat	R00.0, R00.1, R00.2	Ever	785.0, 427.81, 427.89, 785.1
**Chronic kidney disease**			
Lipoprotein deficiency	E78.6	Ever	272.5
Non‐neuropathic heredofamilial amyloidosis	E85.0	Ever	277.31
Rapidly progressive nephritic syndrome	N01*	Ever	580.4
Syphilis of kidney and ureter	A52.75	Ever	095.4
Tuberculosis of kidney and ureter	A18.11	Ever	016.00, 016.20
*Plasmodium malariae* malaria with nephropathy	B52.0	Ever	084.9 with 581.81
Recurrent and persistent hematuria	N02*	Ever	581.1, 581.2, 581.3, 581.89, 581.9
Chronic nephritic syndrome	N03*	Ever	582.0, 582.1, 582.2, 582.4, 582.89, 582.9
Nephrotic syndrome with diffuse mesangial proliferative glomerulonephritis	N04.3	Ever	581.2
Nephrotic syndrome with diffuse endocapillary proliferative glomerulonephritis	N04.4	Ever	581.0, 581.2
Nephrotic syndrome with diffuse mesangiocapillary glomerulonephritis	N04.5	Ever	581.2
Nephrotic syndrome with dense deposit disease	N04.6	Ever	581.2
Nephrotic syndrome with diffuse crescentic glomerulonephritis	N04.7	Ever	581.89
Nephrotic syndrome with other morphologic changes	N04.8	Ever	581.89
Nephrotic syndrome with unspecified morphologic changes	N04.9	Ever	581.9
Unspecified nephritic syndrome	N05*	Ever	583.1, 583.2, 583.89
Hereditary nephropathy ‐ not elsewhere classified	N07*	Ever	583.1, 583.2, 583.89
Glomerular disorders in diseases classified elsewhere	N08*	Ever	583.81
Chronic tubulo‐interstitial nephritis	N11*	Ever	590.00, 590.01, 590.80, 593.3, 593.4
Analgesic nephropathy	N14.0	Ever	583.89
Nephropathy induced by other drugs, medicaments and biological substances	N14.1*	Ever	583.89
Nephropathy induced by unspecified drug, medicament or biological substance	N14.2	Ever	583.89
Nephropathy induced by heavy metals	N14.3	Ever	583.89
Other renal tubulo‐interstitial diseases	N15*	Ever	583.89, 590.2
Renal tubulo‐interstitial disorders in systemic connective tissue disorders	N16*	Ever	583.81, 590.81
Chronic kidney disease	N18*	Ever	585.1, 585.2, 585.3, 585.4, 585.5, 585.6
Disorders resulting from impaired renal tubular function	N25.0, N25.1, N25.81, N25.89, N25.9	Ever	588.0, 588.1, 588.81, 588.89, 588.9
Unspecified contracted kidney	N26*	Ever	587, 405.91
Cystic kidney disease	Q61.02, Q61.11, Q61.19, Q61.2, Q61.3, Q61.4, Q61.5, Q61.8	Ever	753.14, 753.13, 753.12, 753.15, 753.16, 753.17, 753.19
Alport syndrome	Q87.81	Ever	759.89
Hypertensive chronic kidney disease	I12*	Ever	403.00, 403.01, 403.10, 403.11, 403.90, 403.91
Hypertensive heart and chronic kidney disease	I13*	Ever	404.00, 404.01, 404.02, 404.03, 404.10, 404.11, 404.12, 404.13, 404.90, 404.91, 404.92, 404.93
Cloudy (hemodialysis) (peritoneal) dialysis effluent	R88.0	Ever	792.5
Encounter for care involving renal dialysis	Z49*	Ever	V56.1, V56.2, V56.31, V56.32
Dependence on renal dialysis	Z99.2	Ever	V45.11
Abnormal results of kidney function studies	R94.4	Past 1 year	794.4
Sjögren syndrome with tubulo‐interstitial nephropathy	M35.04	Ever	710.2 with 583.81
Patient's noncompliance with renal dialysis	Z91.15	Past 5 years	V45.12
**Diabetes**			
Encounter for fitting and adjustment of insulin pump	Z46.81	Ever	V53.91, V65.46
Presence of insulin pump (external) (internal)	Z96.41	Ever	V45.85
Diabetes mellitus due to underlying condition	E08*	Past 5 years	249.20, 249.10, 249.30, 249.40, 249.50, 249.90
Drug or chemical induced diabetes mellitus	E09*	Past 5 years	249.20, 249.10, 249.30, 249.40, 249.50, 249.60, 249.70, 249.80, 249.00
Type 1 diabetes mellitus	E10*	Past 5 years	250.11, 250.31, 250.41, 250.51
Type 2 diabetes mellitus	E11*	Past 5 years	250.00, 250.10, 250.20, 250.40, 250.50, 250.60, 250.70, 250.80, 250.90
Other specified diabetes mellitus	E13*	Past 5 years	249.10, 249.20, 249.30, 249.40, 249.50, 249.60, 249.70, 249.80, 249.90, 250.10, 250.20, 250.30, 250.40, 250.50, 250.60, 250.70, 250.80, 250.90
**Immunocompromised status**			
*Transplant*			
Transplanted organ and tissue status	Z94*	Ever	V42.0, V42.1, V42.3, V42.4, V42.5, V42.6, V42.7, V42.81, V42.82, V42.83, V42.84, V42.89
Complications of transplanted organs and tissue	T86*	Ever	996.51, 996.52, 996.69, 996.79, 996.85, 996.81, 996.82, 996.83, 996.84, 996.86, 996.87, 996.88, 996.89
*Malignancy*			
Hodgkin lymphoma	C81*	Past 2 years	201.00–201.08, 201.10–201.18, 201.20–201.28, 201.40–201.48, 201.50–201.58, 201.60–201.68, 201.70–201.78
Follicular lymphoma	C82*	Past 2 years	202.00–202.08, 202.80–202.88
Non‐follicular lymphoma	C83*	Past 2 years	200.00–200.08, 200.10–200.18, 200.20–200.28, 200.30, 200.50–200.58, 200.70–200.78, 200.80–200.88
Mature T/NK‐cell lymphomas	C84*	Past 2 years	200.60–200.68, 202.10–202.18, 202.20–202.28, 202.70–202.78, 202.80–202.88
Other specified and unspecified types of non‐Hodgkin lymphoma	C85*	Past 2 years	200.70–200.78, 202.80–202.88,
Other specified types of T/NK‐cell lymphoma	C86*	Past 2 years	202.81, 202.87, 202.83, 202.80
Malignant immunoproliferative diseases and certain other B‐cell lymphomas	C88*	Past 2 years	273.3, 203.80, 203.81, 200.30, 238.79
Multiple myeloma and malignant plasma cell neoplasms	C90*	Past 2 years	203.00–203.02, 203.10–203.12, 203.80–203.82
Lymphoid leukemia	C91*	Past 2 years	204.00–204.02, 204.10–204.12, 204.80–204.82, 202.40, 204.90–204.92
Myeloid leukemia	C92*	Past 2 years	205.00–205.02, 205.10–205.12, 205.20–205.22, 205.30–205.32, 205.80–205.82, 205.90–205.92
Monocytic leukemia	C93*	Past 2 years	206.00–206.02, 206.10–206.12, 206.20–206.22, 206.80–206.82, 206.90–206.92
Other leukemias of specified cell type	C94*	Past 2 years	207.00–207.02, 207.20–207.22, 207.80–207.82, 238.79
Leukemia of unspecified cell type	C95*	Past 2 years	208.00–208.02, 208.10–208.12, 208.20–208.22, 208.80–208.82, 208.90–208.92
Other and unspecified malignant neoplasms of lymphoid, hematopoietic and related tissue	C96*	Past 2 years	202.30, 202.50, 202.60, 202.90–202.92, 277.89
*Immunosuppressive medications*			
Personal history of immunosuppression therapy	Z92.25	Past 6 months	V87.46
Encounter for antineoplastic radiation therapy	Z51.0	Past 6 months	V58.0
Encounter for antineoplastic chemotherapy and immunotherapy	Z51.1	Past 6 months	V58.11, V58.12
*Other immunodeficiencies*			
Functional disorders of polymorphonuclear neutrophils	D71*	Past 10 years	288.1
Immunodeficiency with predominantly antibody defects	D80*	Past 10 years	279.00, 279.01, 279.02, 279.03, 279.04, 279.05, 279.09
Combined immunodeficiencies	D81*	Past 10 years	279.13, 279.2
Immunodeficiency associated with other major defects	D82*	Past 10 years	279.11, 279.12, 279.8
Common variable immunodeficiency	D83*	Past 10 years	279.06
Other immunodeficiencies	D84*	Past 10 years	277.6, 279.8, 279.10, 279.19, 279.3

^a^ Definition of high‐risk conditions were not mutually exclusive. For example, an individual with chronic obstructive pulmonary disease could also have had heart failure. In such a case, that individual would have been counted in both the “chronic obstructive pulmonary disease” category and the “heart failure” category.

^b^ Time frame looked backwards in time beginning with the index date, which September 1 immediately preceding the start of that winter respiratory virus season. For example, for a condition that must be observed within the past 5 years, a participant in the Flu VE study during the 2018–19 season must have at least 1 ICD code observed within the 5 years preceding September 1. 2018.

* Asterisks indicate the inclusion of all constituent (“child”) codes underneath the parent code with the asterisk.
